# 26 Years of Skin Banking in Finland

**DOI:** 10.3390/ebj5040038

**Published:** 2024-12-09

**Authors:** Kaarle Antila, Jyrki Vuola, Andrew Lindford

**Affiliations:** Helsinki Burn Centre, Department of Plastic Surgery, Helsinki University Hospital and University of Helsinki, 00029 Helsinki, Finlandjyrki.vuola@helsinki.fi (J.V.)

**Keywords:** allograft skin, skin bank, glycerol, procurement, organ donor, burns

## Abstract

Autologous skin grafts are the gold standard for definitive wound coverage in burn care, but allograft skin grafts are essential for providing temporary coverage in cases of extensive burns. The Helsinki Skin Bank, established in 1995 at the Helsinki Burn Centre, is Finland’s only licensed skin bank, serving a population of 5.5 million. It procures human skin allografts from multi-organ donors in the Greater Helsinki area and preserves them using glycerol, a method pioneered by the Euro Skin Bank. Between 2009 and 2020, the Helsinki Skin Bank procured skin from 263 donors and provided allografts to 248 patients, primarily burn victims. Over time, procurement methods have improved significantly, resulting in an increase in the amount of skin harvested per donor. Despite rising costs due to more stringent European Union regulations and the need for round-the-clock operations, the bank has remained cost-effective. The glycerol preservation method ensures microbiological safety and effective storage, with minimal contamination issues. The future may see advances in skin substitutes and stem cell treatments, but for now, allogenic skin remains crucial in burn care due to its availability, ease of use, and cost-efficiency. Running a small, professional skin bank for a single burn center has proven successful and sustainable.

## 1. Introduction

The Helsinki Skin Bank was established in 1995 in the Helsinki Burn Centre, belonging to the Department of Plastic Surgery at the Helsinki University Hospital. It is the only licensed glycerolized skin bank in Finland (pop. 5.5 million) as well as in Scandinavia. The Helsinki Skin Bank procures and preserves human skin allografts which are obtained from multi-organ donors from the Greater Helsinki area (1.52 million people). The allografts are preserved using a glycerol-preservation method that was originally developed by the Euro Skin Bank in the early 1980s [1]. The allografts produced by the Helsinki Skin Bank are not commercially distributed or sold. They are primarily procured and utilized to meet the needs of the Helsinki Burn Centre. 

The gold standard for wound coverage in deep dermal and full thickness burn injuries is the use of autologous skin grafts. However, autografts are not always sufficiently available when treating extensive burns, so donor allograft skin is necessary to provide temporary wound coverage in patients with extensive skin loss. First utilized during World War II, cadaveric skin allografts have become a cornerstone of burn management, with their efficacy well-documented and their use is now routine in numerous major burn centers worldwide. Allografts serve as a temporary coverage priming the wound bed, alleviating pain, reducing scarring, preventing infection and maintaining patient homeostasis by reducing loss of fluids, protein and heat through the burn wound. Allograft skin can also be used in a sandwich technique, where allograft skin is placed over widely meshed autologous skin to protect it from mechanical damage, dehydration, and infection. Allografts are used extensively in burn surgery and the importance of local and regional skin banks cannot be overstated [2,3,4].

Skin banking approaches differ significantly between high-income countries (HICs) like Finland and other European nations, and low- and middle-income countries (LMICs) due to disparities in resources, infrastructure, and healthcare priorities. In HICs, skin banks are typically part of well-established networks integrated with regional burn centers. These systems are supported by advanced regulations, high investment in infrastructure, and sophisticated donor programs ensuring quality and safety. LMICs face numerous challenges in developing skin banks. Limited resources often prioritize acute care over the establishment of tissue banking infrastructure. Many countries rely on importing preserved skin from international sources due to the high costs of local banks and insufficient expertise in preservation techniques [5,6].

The most commonly used methods for preserving cadaveric skin are glycerol preservation and cryopreservation. The cells in the glycerol preservation method are non-viable, unlike in the cryopreservation process, where the cells remain viable at least to a certain extent. In both methods, the structure of the skin remains which seems to be more important than the viability [7]. Good results can be obtained by both methods but factors such as the antimicrobial properties of glycerol and its cost-effectiveness may become more influential in the selection of the preservation method. Glycerol preservation requires only refrigeration at standard temperatures (around 4 °C), making it more practical for storage compared to cryopreserved grafts, which require very low temperatures for long-term storage [7,8].

Current legislation in the European Union regarding tissue banking is strict. The European Parliament and Council Directive 2004/23/EC sets standards of quality and safety for the donation, procurement, testing, processing, preservation, storage, and distribution of human tissues and cells. After the first Directive, there have been two amending Directives (2006/17/EC and 2012/39/EU) as regards certain technical requirements for the testing, donating, and procurement of human tissues and cells. The Council of Europe has also published a guide for the quality and safety of tissues and cells for human application, with the latest fifth edition published in 2022 [9].

The early years and setting up of The Helsinki Skin Bank has been described earlier by Vuola et al. [10]. In short, the Helsinki Burn Centre began glycerolized skin banking in June 1995 amidst limited funding, initially striving to end the use of fresh cadaver skin as a temporary coverage and to minimize the costs by obviating the need to purchase allograft skin from abroad. A unique approach in skin procurement was achieved by training operation theater nurses to procure skin from multiorgan donors. 

Over time the scope of allograft skin use expanded to encompass broader indications, including pediatric second-degree scalds and facial burns. Procurement was confined to the Greater Helsinki area for practicality and optimizing the coordination with multiorgan retrieval. 

The period spanning from 2001 to 2008 is detailed in an earlier article by Lindford et al. [11]. Numerous new strategies were successfully implemented including a rotation of 8–10 nurses (2 at a time) for skin procurement resulting in improved yield per donor. In contrast to its early years, the operation became even more professional. The price per square centimeter increased by 32.8% over the span of 7 years (2001–2008), likely due to inflation and the need to comply with evolving regulations and operational standards, which may have increased labor and material costs. Despite this, it remained more cost-effective than externally sourced skin. 

The objective of this current article is to review the management and running of the Helsinki Skin Bank between the years 2009 and 2020. Furthermore, the goal is to determine how the quantity of the allografts has changed over time and which factors have contributed to it. We are also interested in how new protocols and legislation have influenced the running of the Helsinki Skin Bank. Additionally, the objective encompasses the recognition of overall long-term trends since the founding of the skin bank in 1995 until 2020. We included the data from 2004 to 2008 in Section 4 to demonstrate longer-term trends.

## 2. Materials and Methods

The files of the Helsinki Skin Bank were reviewed to identify allograft donors and recipients between 1 January 2009 and 31 December 2020. The following donor data were collected: number of donors per year, donor age, harvested skin area and microbiological findings. Recipient data were collected from an electronic database and comprised the following: number of recipients, recipient age, number of operations, size area of allografts used to treat a recipient, number of donors per recipient, size and cause of the burn injury.

We estimated the total area covered with allograft skin for a recipient by first dividing the total area (cm^2^) of skin collected from the donors by the number (n) of allografts. The average size of an allograft was approximately 113.6 cm^2^. This size was used to calculate the area (cm^2^) of allografts used per recipient, as we only had information on how many allografts were used in operations.

### Harvesting and Processing of the Allografts

The allograft skin is harvested from multiorgan donors from the greater Helsinki area. Procurement and processing of the skin is organized by the plastic surgery theater nurses which is a unique approach in Europe. Approximately 10 nurses are trained for skin procurement. This operation is based on the voluntary activity of nurses who do not have any designated shifts. Instead, a sequentially changing departure order ensures equal opportunities for everyone to take part in the operation. The skin bank has a telephone number that the nurses take turns in answering. The nurse who responds to the call informs other members of the skin bank team about the available donor. Responsibility is then delegated to the skin procurement team which consists of three nurses per multiorgan donor. Prior to 2012, procurement was conducted by only two nurses. Following procurement, the three nurses are then placed at the bottom of the departure order for the next donor operation. Additionally, the nurses assigned to each procurement are responsible for the processing and storage of procured allografts.

Whenever a multiorgan donor becomes available for organ donation the transplant team of the Helsinki University Hospital informs the plastic surgical skin bank nurses of a possible candidate. We follow the “Guide to the quality and safety of tissues and cells for human application” when including and excluding the donors [9]. In short, all donors that are eligible to be multi-organ donors are automatically considered for allograft skin donation. All donors are 18 years or older. Regarding potential cytomegalovirus (CMV) transmission, approximately 70–75% of our multiorgan donors are tested seropositive for previous CMV infection (IgG antibodies). In the very rare case that the donor has a high amount of IgM CMV antibodies as a sign of acute infection, we discard the allografts. Furthermore, glycerol is a well-known antiviral agent, and therefore it is not considered a contraindication to use harvested skin from CMV positive donors [12]. Other more donor skin specific exclusion criteria include systemic autoimmune or connective tissue diseases affecting the skin, systemic corticosteroid use, or any other widespread skin disease affecting the safety or quality of skin. 

After the donor has been cleared for donation by the organ transplantation team, different teams procure all the required organs and tissues. At this point, the blood circulation is discontinued, and the skin bank nurses enter the theater. The donor patient is re-scrubbed and draped. Skin is harvested using an air-driven dermatome. The allografts are procured from the donor’s flanks, limbs, back, and buttocks.

Following procurement, donor skin is divided into smaller batches and immersed in an 85% glycerol and antibiotic solution containing G-penicillin and streptomycin. Subsequently, it is incubated at 38 °C for 3 h. The skin is then transferred under sterile conditions to a solution of 85% glycerol and saline, followed by another 3 h incubation period at the same temperature. In the last step the skin is immersed in 85% glycerol and refrigerated under sterile conditions. Bacterial and fungal cultures are taken after 3 weeks and once the results are negative, the skin is deemed suitable for use. The allografts are divided into smaller batches for the convenience of the surgical team and to ensure aseptic protocols. This reduces the likelihood of using the same container for multiple operations which could lead to cross-contamination.

## 3. Results

Between 2009 and 2020 we identified 263 allograft donors. 119 (45.2%) of the donors were women and 144 (54.8%) were men. The number of donors per year varied from 5 to 32 donors with a mean of 22. Since the year 2012 the number of donors per year has remained at over 20. The mean age of donors varied yearly from 49.7 to 61.6 years with a mean of 55.0. The age has been steadily increasing during the study period. The area of harvested skin per year varied from 11,632 cm^2^ to 179,270 cm^2^ with a mean of 115,230 cm^2^. The harvested area per donor ranged from 1385 cm^2^ to 13,929 cm^2^ with a mean of 5416 cm^2^. The donor data are presented in Table 1.

12 out of 392 batches of allograft skin were discarded during the study period. The reasons included bacterial contamination (n = 6), broken container (n = 3), poor-quality skin (n = 2) and donor was excluded (n = 1).

During the same period 248 patients were treated with allograft skin. The number of allograft recipients varied yearly between 12 and 33 with a mean of 20. The study population had a mean age of 45.5 years, with ages ranging from newborn to 90 years. Among the recipients, 38 were under the age of 18 which comprised 15.3% of all recipients. Among the total patient cohort, 208 (84%) individuals experienced burn injuries, with a mean Total Body Surface Area (TBSA) of 31.9% (range: 0.5–92.5%). The mean number of surgeries per recipient where allograft skin was used was 2.1 with a range between 1 and 27. An average of 5050.5 cm^2^ of allograft skin was used per recipient (range 113.6–41,577.6 cm^2^). Table 2 provides the recipient data. 

**Figure 1:**
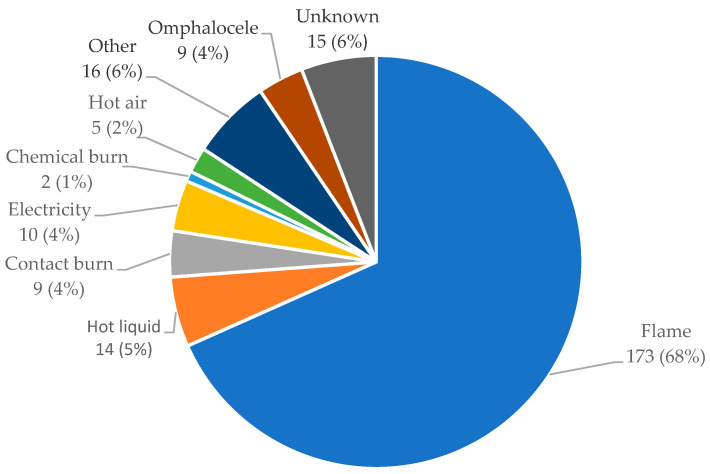
The different causes of injury amongst the recipients between 2009 and 2020 (n = 253). The group ‘Other’ includes the following causes of skin loss/absence: contusion hematoma, lyell’s syndrome, breast cancer, basal cell carcinoma of the head, hip fracture, nasal basal cell carcinoma, trichoblastic carcinoma, sepsis, shin ulcer, pressure ulcer, other wound (one case each), squamous cell carcinoma (2 cases), and necrotizing fasciitis (3 cases).

The different causes of injury/absence of skin amongst the 248 recipients are presented in Figure 1. The most common cause was a burn induced by a flame (68.4%).

## 4. Discussion

The Helsinki Skin Bank began operating 26 years ago with modest organization and low costs. Over time it has developed into a well-functioning glycerolized allograft skin bank with professional harvesting and storing procedures fulfilling all tissue bank regulations as set by the European Union. The Helsinki Skin Bank has made significant advancements in the procurement of high-quality allograft skin since its inception. The mean quantity of skin harvested per donor experienced a more than four-fold increase when comparing the initial period (1995–1999) to the subsequent years (2012–2020), rising from 1400 cm^2^ per session to 5738 cm^2^. Additionally, the amount of annually collected skin has seen a substantial rise over the years, from a mean of 16,382 cm^2^ annually during 1995–1999 to 141,831 cm^2^ annually between 2012 and 2020 [10].

Before 1995, fresh cadaver skin harvested by the Plastic Surgeon on-call, was used in the treatment of large burns in the Helsinki Burn Unit. This approach did not produce enough allograft skin, and the testing of the donors did not meet the standards required for organ donors.

The Helsinki Skin Bank was established in 1995 following a visit to The Euro Skin Bank (Beverwijk, The Netherlands) that distributed glycerol preserved allograft skin across Europe. The glycerol preservation method was ultimately chosen because of the simplicity of the preservation process and acceptable costs of running the bank. The Euro Skin Bank (nowadays Euro Tissue Bank) had a well-functioning operation, and they were very open to share their methods. The Helsinki Skin Bank was established using the same procedures as the Euro Skin Bank. Our skin harvest team has visited the Euro Tissue Bank multiple times over the years, but the glycerol preservation method has not changed substantially although there have been some minor improvements. Procurement was conducted from the very beginning in close coordination with the Transplantation Unit of the Helsinki University Hospital. This ensured that all the donors were deemed suitable for organ donation at the outset by the transplantation unit and that the procurement then took place in an operating theater rather than in a mortuary. Consequently, absence of rigor mortis in donors meant easier handling and movement of the donor. Procurement in an operating theater setting is also less psychologically draining. 

However, eventually it became apparent that the skin banking process needed improvements. The procurement was carried out by the on-call plastic surgeon and some of the donors were missed because the surgeon had to attend emergency operations. Sometimes the on-call surgeon was a resident in the early stages of training unaware of the tricks involved in harvesting skin, or on the other hand, a more senior surgeon lacking motivation during a night shift. In any case this meant that there was not enough procured skin, and the quality varied.

The operation needed a more professional approach, so the Helsinki Skin Bank underwent a reorganizing process in 1999. After the reorganization, a rolling rotation of three trained nurses (one at a time) were available 24 h a day on weekdays for possible allograft procurement. This change increased substantially the amount of allograft skin procured and vastly improved its quality. Prior to the reorganization, a mean of 1400 cm^2^ of skin was harvested per session. Post-reorganization the mean skin yield increased to 3100 cm^2^. Furthermore, only very few donors were missed after the improvements [10].

The development of the operation continued, and in the early 2000s two nurses at a time were deployed to procure skin, leading to improved yield per donor. The microbiological safety of the allografts was deemed excellent since none of the skin batches were discarded due to bacterial contamination [11].

**Figure 2:**
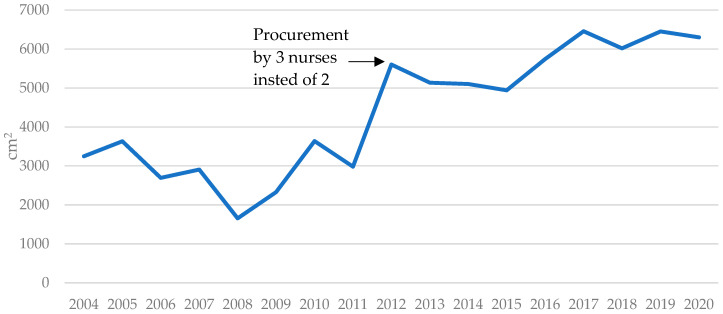
Mean harvest per donor (cm^2^) between January 2004 and December 2020.

**Figure 3:**
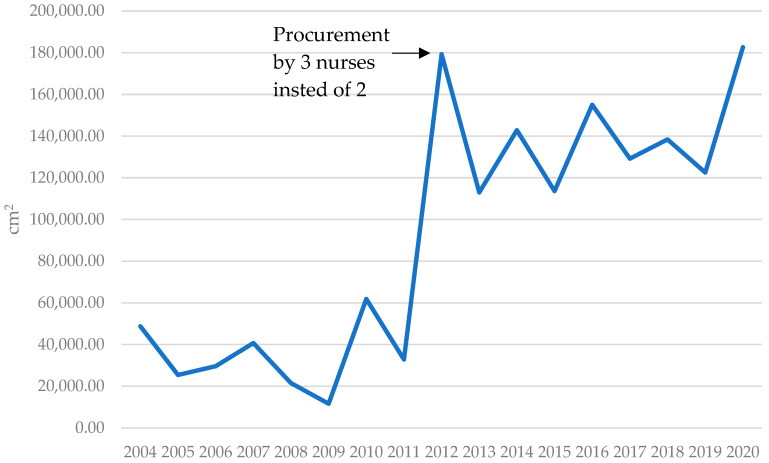
Allograft skin collected from the donors (cm^2^) between January 2004 and December 2020.

In 2012, a third nurse was added to the skin procurement team. The mean harvest per donor increased substantially (2012–2020) to 5738 cm^2^ (range 1562–13,929 cm^2^) per session, as demonstrated in Figure 2. One of the biggest factors for improved yield was the ability of three nurses to turn the patient over and collect skin from the patient’s back and buttocks. Also, prior to 2012 the skin bank nurses were not available for duty at the weekends which resulted in missed donors. Further improvements were achieved by optimizing the deployment of skin bank nurses and the operation became round-the-clock, year-round. Since 2012 the number of donors annually has remained above 20. All the previously mentioned factors have contributed to the annual increase in the total amount of collected skin as demonstrated in Figure 3. 

Since the Skin Bank inception, the costs have increased substantially, at least partially due to the changed EU directives that consider the skin bank to belong under the legislation of tissue banking. The legislation directs the donation, procurement, testing, processing, preservation, storage and distribution of human derived tissues and cells [9]. It requires a lot of documentation and bureaucracy, which increases the workload for the skin bank team. Also, the fact that in 2012 the operation became round-the-clock, year-round, and a third nurse was added to the skin procurement team increased the total labor hours. In fact, a significant portion of the rising costs can be attributed to the increased labor costs due to extended effort and higher wages. Still, the benefits of having a locally run skin bank are worth the investment.

The low rate of discarded batches due to bacterial contamination (1.5%) during the study period indicates that the glycerol preservation method is effective. The glycerol preservation method is known to be slow but efficient: 97% of bacteriological cultures test negative within three months [13,14]. The addition of antibiotics during processing enhances the inactivation of microorganisms. Glycerol can also inactivate viruses. HIV-1 was effectively inactivated by 85% glycerol within one hour at 37 °C. Similarly, storing cadaver skin infected with HIV-1 in 85% glycerol at 4 °C led to complete virus inactivation after five days [15]. In our experience, allograft skin is very effective in preventing wound contamination and infection by temporarily closing the wound. This property of allograft skin has been particularly useful in the treatment of war victims with wounds that are highly contaminated with multi-resistant microbes.

Despite the three-decade long presence of various burn care products on the market, allogenic skin has maintained its pivotal role. Advancements in skin substitutes and stem cell research will probably replace allogenic skin in the future [16]. Dermal substitutes are utilized in burn care to support the preparation of the wound bed before applying autologous skin grafts, but the high prices are still a major barrier to more widespread use. Additionally, excessive scar formation accounts for major morbidity and remains a continuing challenge in burn treatment and is not directly improved by allograft use [17]. New skin substitutes will at least solve part of these problems, but further research is needed to investigate long-term outcomes, treatment protocols, complications and cost-effectiveness, despite promising preliminary results [18,19,20,21].

Allogenic skin remains essential in burn care due to its ease of processing, handling and storage, as well as its cost-effectiveness, ensuring its consistent availability. Furthermore, having a locally run skin bank significantly enhances its accessibility. By providing a reliable and sustainable source of skin, skin banks contribute to the effective management of burn patients [22,23,24].

## 5. Conclusions

The Helsinki Skin Bank has evolved since its establishment in 1995, improving the procurement, processing, and availability of glycerol-preserved allografts. Key advancements, such as the introduction of a rotating team of trained nurses and round-the-clock operations, have significantly increased the quantity and quality of harvested skin. The glycerol preservation method has proven highly effective, with minimal contamination and high microbiological safety. Despite rising costs, the locally run skin bank remains cost-effective and essential in burn care, ensuring reliable access to allografts for burn victims. Allograft skin continues to play a vital role in treating extensive burns.

## Figures and Tables

**Table 1:** Annual data of the skin donors.

Year	2009	2010	2011	2012	2013	2014	2015	2016	2017	2018	2019	2020
Donors (n)	5	17	11	32	22	30	23	28	23	23	20	29
Donors mean age (years)	51.2	58.1	53.4	49.7	54.2	54.3	53.9	58.6	55.2	54.6	61.6	55.3
Harvested skin (cm^2^)	11,632	61,845	32,795	179,270	112,980	142,816	113,609	155,022	129,169	138,402	122,542	182,673
Mean harvest per donor (cm^2^)	2326	3638	2981	5602	5135	5101	4940	5742	6458	6017	6450	6299
Discarded batches (n)	-	-	-	4	-	2	-	1	3	-	1	-

**Table 2:** Annual data of the recipients.

Year	2009	2010	2011	2012	2013	2014	2015	2016	2017	2018	2019	2020
Recipients (n)	15	20	12	17	22	18	27	33	24	18	19	23
Recipients mean age (years)	48.2	39.9	47.1	42.5	44.4	44.6	50.0	47.8	42.5	50.9	35.6	52.4
Allografts used (cm^2^)	25,106	63,502	61,798	66,115	140,296	109,056	127,232	196,869	94,515	127,459	117,462	92,811
Mean TBSA (%)	26.9	35.6	37.8	49.5	35.1	31.3	31.4	26.2	25.7	31.8	31.3	29.5
Number of operations (n)	20	30	26	43	56	38	53	67	42	67	48	42

## Data Availability

The data presented in this study are available on request from the corresponding author due to privacy reasons.
